# Wild fish consumption can balance nutrient retention in farmed fish

**DOI:** 10.1038/s43016-024-00932-z

**Published:** 2024-03-20

**Authors:** David F. Willer, Richard Newton, Wesley Malcorps, Bjorn Kok, David Little, Anneli Lofstedt, Baukje de Roos, James P. W. Robinson

**Affiliations:** 1 Department of Zoology, University of Cambridge, Cambridge, UK; 2 Institute of Aquaculture, Faculty of Natural Science, University of Stirling, Stirling, UK; 3 The Rowett Institute, University of Aberdeen, Aberdeen, UK; 4 Lancaster Environment Centre, Lancaster University, Lancaster, UK

**Keywords:** Agriculture, Marine biology

## Abstract

Wild fish used as aquafeeds could be redirected towards human consumption to support sustainable marine resource use. Here we use mass-balance fish-in/fish-out ratio approaches to assess nutrient retention in salmon farming and identify scenarios that provide more nutrient-rich food to people. Using data on Norway’s salmon farms, our study revealed that six of nine dietary nutrients had higher yields in wild fish used for feeds, such as anchovies and mackerel, than in farmed salmon production. Reallocating one-third of food-grade wild feed fish towards direct human consumption would increase seafood production, while also retaining by-products for use as aquafeeds, thus maximizing nutrient utilization of marine resources.

## Abstract (web summary)

A mass-balance and fish-in/fish-out ratio approach demonstrates that redistributing wild-caught fish used in salmon aquaculture to human consumption could improve nutrient retention and support sustainable marine resource use.

## Main

Aquaculture expansion is expected to meet increases in global seafood demand and to contribute towards addressing growing malnutrition^1–4^. Both wild and farmed seafood can play an increasingly important role in addressing dietary deficiencies globally, including iodine, iron, omega-3 fatty acid, and vitamin A, D and B_12_ deficiencies^5–8^. Freshwater aquaculture in particular has improved food security in many parts of the world and remains a critical sector^3,9^. However, global demand for marine-fed carnivorous species such as Atlantic salmon (*Salmo salar*) is also growing, directed towards high-income, food-secure countries, but also increasingly to affluent consumers in low- and middle-income countries^10–15^.

Salmon has one of the most efficient feed-to-food conversions among farmed animals, but its high trophic level makes it a resource-intensive food^16^. Growth in salmon production has continued after the supply of marine ingredients (fishmeal and fish oil) peaked, but despite some redress through increased use of fishery by-products, aquaculture has consumed an increasingly large share, now 70%, of this finite resource^2^. Increasing marine ingredient costs and sustainability awareness^17^, improved farm management and better feeding practices have reduced inclusion rates of marine ingredients in salmon feeds and driven greater resource efficiencies over the past two decades^12^. Nevertheless, salmon aquaculture remains a major consumer of marine ingredients from wild fish, including species that are consumed directly by people (for example, herring and mackerel). Use of wild fish species, if directly edible, in salmon feed might therefore be expected to decrease the overall amount of nutritious seafood. A better understanding of nutrient retention from wild to farmed fish is therefore key to improving both marine resource use efficiency and nutritious seafood supply^18–22^.

A range of metrics have been used to assess feed efficiency in aquaculture, typically from the perspective of dependence on wild-caught fish (for example, ‘FIFO’, the ratio of wild ‘fish in’ to farmed ‘fish out’; Methods). FIFO has been used to assess sustainability of feed use^12^ and to account for by-products generated from processing, highlighting socio-economic drivers of feed resources^23^. Our understanding of the proportion of essential dietary micronutrients present in wild fish and fed to farmed fish that are retained for human consumption is still limited^2,5,7,24,25^. Measuring the amount of nutrients in edible portions of wild fish that are converted to feed ingredients but are also directly consumed and marketed as seafood (hereon ECM feed fish), relative to the amount of nutrients in the salmon fillet produced, can provide insights into the nutritional performance of fish farming from a food system perspective.

Here we use Norwegian farmed salmon industry data to estimate edible nutrient retention—the proportion of nutrients in ECM feed fish retained in farmed salmon fillets (Methods)^12,26^—and use this metric to assess pathways towards increasing edible seafood supply. We focus on nine nutrients that are essential in human diets and concentrated in seafood (iodine, calcium, iron, vitamin B_12_, vitamin A, omega-3 (eicosapentaenoic acid (EPA) and docosahexaenoic acid (DHA)), vitamin D, zinc and selenium) and estimate nutrient retention using a mass-balance approach that avoids double counting of processed feed fish. We then assess how ECM feed fish can contribute to UK diets and simulate the effects of increasing edible nutrient retention on seafood supply and by-product upcycling.

## Results

### Nutrient retention

**Fig. 1:**
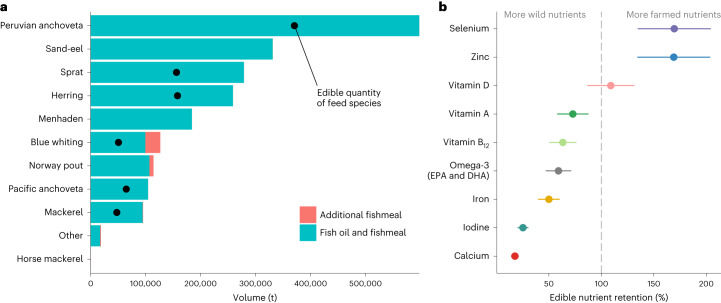
Species composition and nutrient volumes contained in feeds used by Norwegian farmed salmon. **a**, Species composition in fish oil and fishmeal produced by a major feed company, applied to the total volume used to produce Norwegian farmed salmon in 2020. Black points indicate the volume of edible species used to produce fishmeal and fish oil, corrected for edible portions^21^. **b**, Edible nutrient retention for nine nutrients concentrated in seafood and essential for dietary health. Values less than 100% indicate nutrients that have higher yields in ECM fish species (edible portion of consumer-marketed feed fish) than in farmed salmon, based on a mass-balance approach fitted to feed production values in **a**. Uncertainty intervals show variation in retention depending on the edible portion of farmed salmon (58–88%, midpoint = 73% (ref. ^26^)). Source data

Using species-specific fishmeal and fish oil yields^27^, we calculated that Norway’s salmon sector fish oil and fishmeal usage in 2020 required 2,111,283 t of whole fish to be reduced into marine feeds (Fig. 1a and Supplementary Table 1). Of ~2 Mt of whole fish reduced into marine feeds, 40% were ECM species (Fig. 1a). Of these six ECM species, Peruvian anchoveta (*Engraulis ringens*) were the largest contributors to feed (~600,000 t total), used entirely for fish oil in salmon diets, followed by European sprat (*Sprattus sprattus*), Atlantic herring (*Clupea harengus*) and Atlantic mackerel (*Scomber scombrus*) (Supplementary Table 2). Accounting for the quantity and nutrient concentrations of the ECM feed fish (Supplementary Table 3), we have found that these six feed species contained a greater, or similar, concentration of nutrients as farmed salmon fillets (Supplementary Tables 4 and 5), leading to less than 100% nutrient retention for six of nine nutrients in the salmon fillet (Fig. 1b). Quantities of calcium and iodine were, respectively, over five and four times higher in ECM feed fish than in salmon (18% and 25% retention). Quantities of iron, omega-3, vitamin B_12_ and vitamin A were over 1.5 times higher in edible portions of feed fish (<75% retention), whereas quantities of vitamin D were comparable between salmon and ECM feed fish. Zinc and selenium had retention values over 100% (Fig. 1b), indicating that salmon contained a greater quantity of these nutrients than ECM feed fish; the extra zinc and selenium would have been derived from other terrestrial salmon feed ingredients. Thus, while farmed salmon enhanced provision of some nutrients, it limited provision of a greater number of nutrients, leading to net negative nutrient retention.

**Fig. 2:**
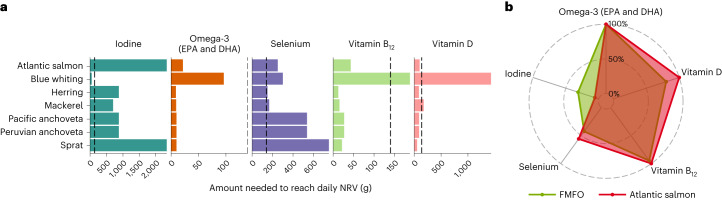
Nutritional composition of edible fishmeal and fish oil species and farmed Atlantic salmon. **a**, Portion size required to reach recommended nutrient intake (NRV) for each ECM fish species and farmed salmon. The dashed line indicates the recommended seafood portion in the United Kingdom (140 g). **b**, Contribution of a 140 g portion to recommended intakes of nutrients in **a**. FMFO, fishmeal and fish oil. Nutrient reference guidelines are for adult women (19–64 years old) (see Extended Data Fig. 2 for male NRVs). Contributions to intakes are capped at 100%, and nutrients with low concentrations or contributions to dietary intakes are not shown (see Extended Data Figs. 1 and 2 for concentrations and recommended intakes for all nine nutrients). Source data

**Extended Data Fig. 1:**
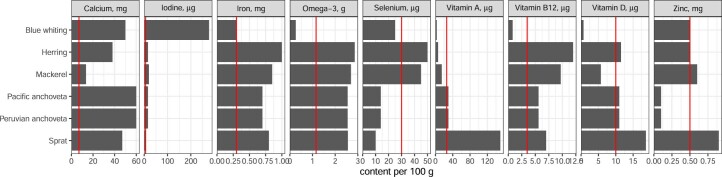
Nutrient content of muscle tissue in edible feed fish species. Red lines show nutrient values for farmed Atlantic salmon.

**Extended Data Fig. 2:**
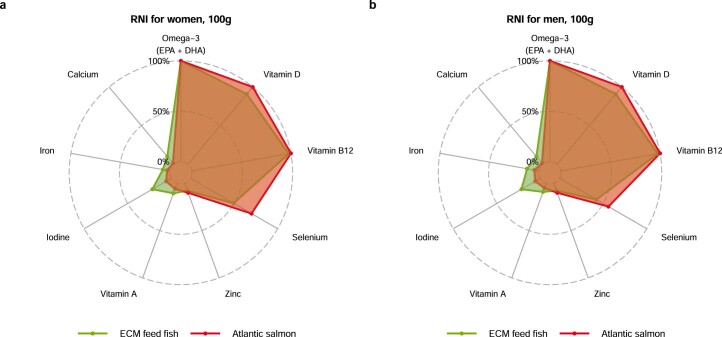
Contribution of 140 g portion to recommended nutrient intakes for the average ECM feed fish (green) and Atlantic salmon (red), for a) adult women (19–64 years old) and b) adult men (19–64 years old). Contributions capped at 100%.

Of the 11 species reduced into marine ingredients for Norwegian salmon in 2020, 6 are available in European markets for direct consumption (Fig. 1a). Most ECM feed fish had similar or greater nutritional value than farmed salmon fillet (Supplementary Table 3), whereby a 140 g portion of both Atlantic salmon fillet and the average ECM feed fish was a dietary source of selenium (>30% of the recommended intake), vitamins B_12_ (>90%) and D (>78%), and omega-3 fatty acids (>100%) (Fig. 2b). However, ECM feed fish met recommended daily intakes of iodine, omega-3 fatty acids (DHA and EPA) and vitamin B_12_ at smaller portions than farmed salmon (Fig. 2a) and also had higher iodine concentrations (140 g of average feed fish provided one-third of dietary iodine requirements; 6% of recommended iodine intake from 140 g salmon portion). ECM feed fish also had higher concentrations of other essential micronutrients, including calcium, iron and vitamin A, than Atlantic salmon, but a 140 g portion did not meet recommended nutrient intakes (Extended Data Figs. 1 and 2, and Supplementary Table 3).

### Directing edible feed species for human consumption

**Fig. 3:**
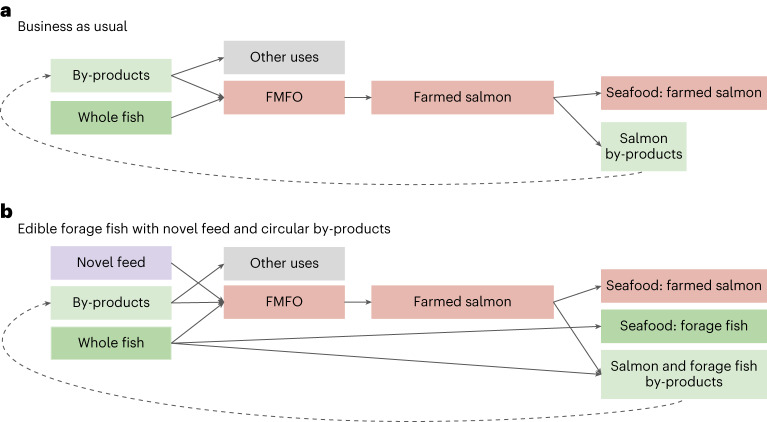
Conceptual overview of the use of whole fish and by-products in farmed salmon production. **a**, Business as usual produces fish oil and fishmeal that are used for salmon feed and other purposes (for example, pet food, export, direct consumption). By-products from salmon may be used to produce more feeds. **b**, We simulate allocation of edible forage fish species (for example, herring, mackerel) for direct human consumption, requiring additional fishmeal and fish oil to maintain salmon production (for example, novel feed) and producing additional by-products. Note that salmon by-products cannot be used to produce salmon feed.

**Fig. 4:**
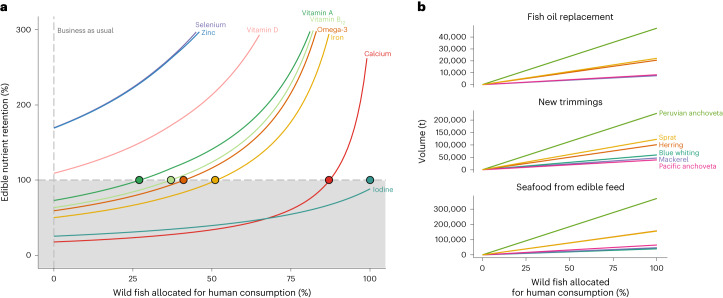
Increasing seafood and by-product output under scenarios of whole-fish direct consumption. **a**, Change in edible nutrient retention as more feed fish are allocated for direct human consumption. The colours indicate nutrients, with edible nutrient retention calculated at business as usual (that is, Fig. 1b, 0% whole fish consumed directly), increasing to 100% of edible whole fish consumed directly. The points show where reallocation of feed fish achieves edible nutrient retention equal to 100%. **b**, Fish oil deficit, new trimmings from edible species that have been consumed (that is, inedible portions) and new seafood produced from direct consumption (that is, edible portions). The lines are different edible species (blue whiting, herring, mackerel, anchoveta, sprat). In each simulation, edible nutrient retention was the total nutrients in seafood (edible portion of salmon and feed fish) divided by the total nutrients in wild fish required to produce fishmeal and fish oil. Source data

We simulated the effect of allocating edible feed species for human consumption, estimating additional fish supply (wild and farmed fish) and associated by-products (that is, trimmings and inedible components) and replacement fish oil or appropriate non-fish alternatives required to maintain salmon production (Fig. 3). The simulations estimate the potential new availability of edible fish and associated by-products, accounting for differences in edible portions between feed fish species. Nutrient retention increased as more feed fish were allocated for direct consumption, reflecting the use of these nutrients as human food rather than as salmon feed (Fig. 4). Direct consumption of 27–51% of ECM fish used in Norwegian salmon feed in 2020 raised nutrient retention above 100% for vitamin A, vitamin B_12_, omega-3 (DHA and EPA) and iron, and improved nutrient retention for calcium and iodine. Selenium, zinc and vitamin D had positive nutrient retention values under the business-as-usual scenario (that is, no direct consumption of ECM fish) and reached over 300% retention at 46–66% feed fish consumption. Our simulations show that relatively small increases in direct consumption of whole fish currently allocated for marine feeds can lead to substantial increases in nutrient retention.

Diverting species currently used for marine ingredients towards human consumption reduced the amount of fish oil available for salmon production, but this fish oil deficit can partially be addressed by using by-products from ECM fish processed as seafood. We next examined trade-offs between seafood production and fish oil supply by removing each ECM species from feed input and assessing change in fish supply and by-products. Anchoveta accounted for most of the fishmeal and fish oil production (Fig. 1a), and so, removing anchoveta from feeds produced the most fish for direct consumption (Fig. 3b) but required a large amount of fish oil replacement (Fig. 3b). By contrast, allocating 100% of mackerel in feed for human consumption produced 47,321 t of seafood (edible portion = 50%; Supplementary Table 3) but required less than 5% additional fish oil accounting for by-product utilization (7,509 t) to maintain 2020 salmon production levels (Fig. 3b). Seafood processing of mackerel redirected to human markets would theoretically still retain 47,321 t of by-products (that is, the inedible portion), which could partially replace marine ingredient supply. Across all species, allocating 100% of ECM fish currently used as feed for human consumption would require an additional 69% of fish oil, either from new sources^28–30^ or from more effective by-product usage. Redirecting feed fish for human food could produce 600,000 t of by-products for use as marine ingredients.

**Extended Data Fig. 3:**
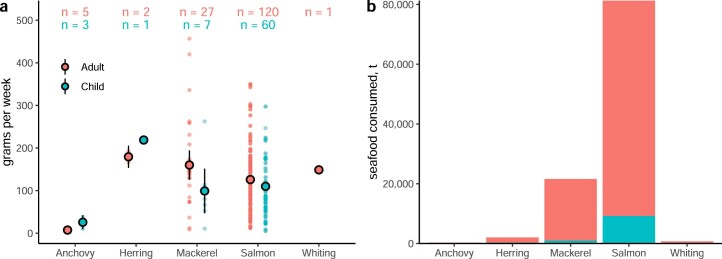
UK seafood consumption reported by National Diet and Nutrition Surveys in 2019. Weekly average consumption reported by species for adults 19+ years old, red) and children (1.5–18 years old, blue) in a, annotated with number of respondents per 500 surveyed people (1000 total). bold points are mean values and bars show 2 standard error from the mean, underlaid with raw values. Data were combined with UK census data to estimate the total annual seafood consumption per species (tonnes) in b, for adults (red) and children (blue).

Our analysis assessed food system changes arising from allocating ECM feed fish for direct human consumption. The demand for and appeal of ECM feed fish are often lower than those for farmed salmon, suggesting that most of these species are unlikely to enter every seafood market as food instead of feed. National diet and nutrition surveys^31^ in the United Kingdom estimate that 24% of adults (12% of children) consumed salmon weekly, exceeding the reported consumption of mackerel (5.4% and 1.4%), herring (0.4% and 0.2%) and anchovy (1% and 0.6%) (Extended Data Fig. 3). Other feed species (for example, sprat) were infrequently reported in 4 day consumption surveys. Low feed fish consumption is also typical of major fishing nations such as Peru and South Africa, where species such as anchovies are primarily used as feed rather than food^32^. Our results suggest that, in the United Kingdom, mackerel is more likely to increase in consumption than other feed species. We find that reallocating one-third of mackerel currently used in the feed of Norwegian-reared salmon for direct consumption would support a 66% increase in annual mackerel consumption in the United Kingdom. By contrast, reallocating just one-third of herring and anchovy used in feeds would far exceed current seafood consumption rates (2,351% and 39,349% of 2019 consumption, respectively).

## Discussion

Our analyses of edible nutrient retention show how reallocating species currently used in salmon feeds to direct human consumption can increase the overall quantity of nutritious seafood, without increases in wild-caught fish supply, while still providing marine ingredients. ECM feed fish contained more nutrients than farmed salmon fillet, with nutrient retention under 100% for six of the nine nutrients indicating nutritional inefficiency of business-as-usual feed use in salmon farming. However, increased human consumption of ECM feed species risks loss of nutrients through seafood processing, suggesting that processing seafood by-products for fish oil and fishmeal production can also help to maximize nutrient retention.

Salmon aquaculture is considered a net-neutral producer of fish biomass, but our results show that it is a net consumer of nutrients available in wild-caught, edible feed fish. Most ECM fish met dietary nutrient recommendations at smaller portion sizes than Atlantic salmon, including omega-3 fatty acids (0.25 g EPA and DHA for women between 19 and 64 years old) and vitamins B_12_ and D (1.5 and 10 g d^−1^, respectively). Farmed salmon is promoted for its omega-3 content, which can reduce the risk of cardiovascular disease and stroke, and increase life expectancy^33^, yet most edible feed fish contained higher concentrations of omega-3 fatty acids than salmon. ECM fish also contained higher levels of iodine, calcium, iron and vitamin A than farmed salmon. Dietary deficiencies of these micronutrients are prevalent across the world^34^. In the United Kingdom, for example, 71% of adults have insufficient vitamin D in winter (38% in summer)^35^, 70% of 14–15-year-old girls have mild to moderate iodine deficiency^36^ and 15–49-year-old women have insufficient selenium (50%) and iron (21%) intakes^34,37,38^. Increasing consumption of feed species could hence contribute to efforts in reducing population-wide nutrient deficiencies such as iodine and vitamin D deficiencies^39,40^. Efforts to increase feed fish consumption by people will, however, depend on consumer demand and market supply, with current consumption of ECM feed fish considerably lower than that of farmed salmon. In the United Kingdom, 80,000 t of salmon was consumed in 2019, far outweighing the 20,000 t of mackerel and less than 3,000 t of herring, anchovy or whiting (Extended Data Fig. 3). Mackerel was consumed most frequently of all ECM species (5% of mackerel-consuming adults, 160 g week^−1^), and thus, within the current UK seafood systems, this species probably has the greatest potential for increased direct consumption. Indeed, from a nutritional perspective, farmed salmon have been popularized in part for their high omega-3 content, suggesting that oily ECM feed species, such as mackerel and herring, could similarly be marketed for health outcomes. Mackerel are also relatively minor contributors to marine ingredients, accounting for just 4% of fish oil used in Norway in 2020, suggesting a potential for large increases in mackerel consumption without impacting fish oil supply.

Low levels of ECM feed fish consumption suggest that there is a need to promote diversity in seafood diets^41^. Policy efforts might draw on the greater affordability of feed fish species than farmed species (for example, equivalent edible yield herring and mackerel <£8 kg^−1^ in 2021, salmon £16 kg^−1^ (ref. ^42^)). However, low consumer prices for ECM species in Europe drive their export for food and feed^43,44^. For example, in 2016, 30% of the £916 million landings from the UK fishing fleet were landed abroad in pursuit of higher market prices for marine ingredients and whole fish^45^, and also for food where European pelagics are sold to West Africa and Asia^46^. Trade policies are thus key influences on demand for marine fish and public consumption patterns^44,47,48^, requiring transformation of global trade systems to protect ECM feed fish where they are locally consumed (for example, mackerel in the United Kingdom or sardinella species in West Africa^49^).

Building consumer demand for feed fish products will be a key component in efforts to improve the use of edible marine resources. ECM feed fish are associated with unappealing and stronger taste, processing difficulties (for example, bones, skin), inconvenience for consumers and lack of consumer knowledge^50^. Processed fillets from larger species are convenient, boneless and easy to prepare (for example, ‘big five’ of cod, tuna, haddock, salmon and prawns), and comprise 80% of all seafood consumed in the United Kingdom^50,51^. This type of fillet is less popular and less widely available from ECM feed fish^42^, though feed fish such as sardines, mackerel and anchovies are more widely and cheaply available as canned products^52^. Canning typically has a larger edible fraction and softens nutrient-rich skin and bones, making canned feed fish particularly nutritious^52^. However, canned tuna, which is skinless and boneless, remains more popular than these products^42,53^, and fresh fish consumption still far outstrips canned fish consumption in high-income countries^54^. Improving convenience, visual exposure and appeal, and flavour profiles through food processing could all help to increase feed fish demand^55,56^, as shown by the rising popularity of frozen fish^50^. A greater variety of products made from ECM feed fish, such as battered sardines, mackerel fishcakes, fishloaf and other ready meals containing a mix of species, could align with national dietary recommendations to improve oily fish consumption^57^. Major demand change is feasible; campaigns such as Sainsbury’s ‘Switch the Fish’ campaign in 2011 were linked to increases in mackerel sales^58^, and product innovation could lead to new increases in ECM fish provisioning to consumers as more species and cuts of feed fish become widely marketed.

To enhance nutritious seafood supply by consuming feed fish as seafood, policies must also promote the use of processing by-products for marine ingredients (Fig. 3b). For example, the processing of mackerel currently used for ECM would supply 47,321 t of usable nutrient-dense by-products, such as skin, viscera, heads and bones^27^. Marine ingredients from by-products are generally considered to be less environmentally impacting than those from whole fish because of the historical high wastage of fish by-products^23,26,27,59,60^, and full by-product utilization can potentially more than replace whole fish required for current fishmeal and oil production^61^. Consequently, upcycling by-products from edible seafood processing for use in feeds is essential from both nutritional and environmental perspectives. In 2022, around 35% of fishmeal and fish oil was produced from processing by-products (an increase from 8% in 2000)^27^, suggesting that increased by-product utilization is already helping the transition towards resource-efficient food systems^62^.

Reducing use of fish oil in aquaculture will require alternative ingredients rich in polyunsaturated fatty acids (EPA and DHA). Conventional vegetable oils are not a suitable replacement, containing high levels of omega-6 and low levels of plant-based omega-3 fatty acids^4,18^. Indeed, farmed salmon now contain less omega-3 and a higher omega-6 to omega-3 ratio than 10 years ago owing to a reduction in fish oil and an increase in vegetable oil use in feeds^20^. At present, algal oils offer the most promising alternative; for example, recent studies show algal oils can completely replace fish oil in shrimp feed, with no difference in growth or feeding efficiency versus a fish oil diet^63^. There are now commercial operations producing *Schizochytrium* sp. oil that is more than 50% polyunsaturated fatty acids (PUFAs) and, unlike other algal oils, is a mix of both EPA and DHA. *Schizochytrium* sp. oil can be an effective source of omega-3 for salmonids^28^ and can be grown heterotrophically using sugar cane or beet by-products. There are trade-offs in environmental performance and cost compared with marine ingredients through high-energy consumption, but performance continues to improve and production is scalable^30,64^. Other ‘novel’ alternatives to fish oil, such as yeast oil or genetically modified omega-3 plant oils^28^, are also entering the market. Novel oil aquaculture industries, as with any small-scale early-stage industry, may face financial barriers and other challenges in competing with established, large-scale marine ingredient industries for market share. There is therefore a need for investments and market incentives to help reduce this perceived ‘green premium’ and promote sustainable growth^29^.

There is still further opportunity to build on nutrient retention analyses. Our mass-balance approach to estimating nutrient retention is a back-calculation of salmon production values in one country in one year that is not currently generalizable to other fed-aquaculture systems. For example, we were unable to account for nutrient yields in spare fishmeal (although fishmeal composition suggests that this is more concentrated in calcium and iodine than omega-3 fatty acids (Supplementary Tables 3 and 4)). Mass-balance approaches also fail to account for fish oil being the limiting marine ingredient, and how feed resources and by-products contribute to food production in a broader sense (for example, livestock). Developing life-cycle approaches that capture the ratio of nutrients in to nutrients out (for example, a ‘nutritional FIFO’, or ‘nFIFO’), and integrating these with economic factors (for example, eFIFO), will ensure that all nutrient flows are considered appropriately^23^. Developing a robust and generalizable method for such an nFIFO metric will help industry to assess, compare and improve nutrient efficiency in farm production, as shown by the wide adoption of FIFO and the forage fish dependency ratio (FFDR) for salmon certification schemes^53^.

In conclusion, assessing nutrient retention from feed to farmed seafood is a useful tool to drive better performance of aquaculture and identify pathways towards sustainable growth. Farmed salmon has succeeded in providing nutritious products in formats popular with customers and plays an important role in UK diets, but the sector needs to improve its practices to grow sustainably^65^. The salmon industry, including feed producers and farm systems, must focus efforts on improving nutrient retention from feed fish to salmon products. The development of nFIFO methodologies based on life-cycle accounting of nutrients in feed–seafood flows such as used in ‘eFIFO’^23^ will help to operationalize this framework for industry purposes. The interventions we propose to bring nutrient retention closer to 100% will more efficiently use finite marine-ingredient resources and provide more people with essential dietary nutrients from a diverse selection of fish. Efficient by-product utilization, new fish oil alternatives, improved infrastructure and regulation, and innovating a range of affordable, appealing and convenient food products using ingredients from feed fish will be critical. These approaches can help bring us towards sustainable, fish-inclusive diets, containing a diverse ‘basket’ of different fish species in which nutrition, economic outcomes and environmental sustainability are all carefully balanced.

## Methods

There are several approaches to estimating FIFO in aquaculture. A previous study calculated whole fish used in fishmeal and fish oil production separately^60,66^, but this can lead to a double counting of fish resources^59,60,66^. Another study updated this to combine fishmeal and fish oil inclusion and yields, equally distributing the whole fish used depending on the mass yield of fishmeal and fish oil, avoiding double counting^61^. This obscures the effect of growing demand for fish oil as a limiting ingredient, potentially increasing the pressure for wild-caught fish, leading to the development of the eFIFO. eFIFO uses economic allocation to assign the whole fish to fishmeal and fish oil depending on the value of both ingredients, typically allocating more whole fish to the more valuable and limiting fish oil ingredient^16^. In this way, economic value acts as a proxy for the nutritional value of ingredients^23,67^. As different species have varying requirements for fishmeal and fish oil, the dietary inclusion rarely matches the proportions from rendering resulting in ‘spare’ ingredients that may be used elsewhere. Early FIFO calculations did not account for this, and FFDRs still use this method. The eFIFO method avoids double counting of these ‘spare’ wild fish resources for aquaculture production and includes by-product resources that other methods do not. Other metrics focus specifically on whole fish use, such as the FFDR that measures the total volume of wild, whole fish required to produce the fishmeal or fish oil^67^. However, eFIFO and FFDR still do not consider the nutritional efficiency of using wild fish as feed for aquaculture^18^. The marine nutrient dependency ratio measures the conversion of marine protein and oils in feed fish to farmed fish, offering some assessment of nutritional efficiency on a macronutrient level^12,67^, but is often based on feed nutritional quality rather than on the species reduced into feeds. In this study, we estimated ‘edible nutrient retention’, focusing on nutrients that are essential to human health and concentrated in seafood. We adopted a mass-balance approach to quantify the edible volume of nutrients in feed species and in farmed salmon fillets.

We collected recently published data on feed composition, fishmeal and fish oil usage, and salmon production in Norway. In 2020, Norway produced 1,467,655 t of Atlantic salmon, using 203,597 t of fish oil and 239,711 t of fishmeal^65^. Using species-specific estimates for fishmeal and fish oil yields from whole fish and trimmings^27^, we estimated the total volume of whole fish required to produce Norwegian salmon in 2020, based on fishmeal and fish oil usage (Supplementary Table 1). These values were then combined with feed species composition estimates from a major feed producer^27,68^ to estimate the volume of each fish species reduced into fishmeal and fish oil (Supplementary Table 2). We assumed fish oil was the limiting ingredient, so we estimated the total volume of wild, whole fish required to produce fish oil volumes used in 2020. This volume of whole fish also accounted for most of the fishmeal used in 2020, and we estimated additional fishmeal that was required for five feed species (Supplementary Table 2). Our analysis does not consider how 64,621 t of ‘spare’ fishmeal was used, as this was beyond the boundary of our case study. Feed data were extracted from Skretting, one of the four largest feed companies that supply Norwegian salmon aquaculture^69^. We assumed that their fishmeal and fish oil species composition was representative of fishmeal and fish oil produced by the other feed companies (for example, Biomar, Cargill and MOWI). We note that sardine species were not included in Skretting feeds in 2020, but may have been included in other years, and can account for a large proportion of feed produced by other companies^70^.

We used a mass-balance approach to calculate edible nutrient retention for Norwegian farmed salmon in 2020, defined as the change in edible nutrient yield from feed to salmon^71^:

1 $$\mathrm{{Edible}\,{nutrient}\,{retention}}=\left(\mathrm{{Salmon}\,{nutrients}}/\mathop{\sum }\limits_{j=1}^{n}{\mathrm{Conc}}_{{ij}}\times {\mathrm{Feed}}_{j}\right)\times 100$$

This metric was calculated for each nutrient *i*, using the concentration (Conc) of nutrient *i* in the ECM feed fish species *j*, and volume of species *j* before reduction into feeds (Feed), where Salmon nutrients is the total volume of salmon fillets produced from that feed multiplied by the concentration of nutrient *i* in the salmon fillet. Our approach is based on the nutrient concentrations in the edible portion of each feed species and only considers species that are reduced into feed as ‘whole fish’ but are also marketed for direct human consumption (anchovies, herring, mackerel, sprat, blue whiting) (Supplementary Table 3)^21^ We considered only fishmeal and fish oil that were produced from whole fish, as these were most likely to be suitable for human consumption (that is, excluding fishmeal and fish oil produced from fish by-products, or ‘trimmings’). We regarded sand-eel (*Ammodytes marinus*), menhaden (*Brevoortia patronus*) and pout (*Trisopterus esmarkii*) as currently ‘inedible’ given the non-existent direct consumption market at present. Atlantic salmon production volume was also corrected for edible portion, using the range of values identified in a previous study^26^ (58–88% and midpoint = 73%).

**Extended Data Fig. 4:**
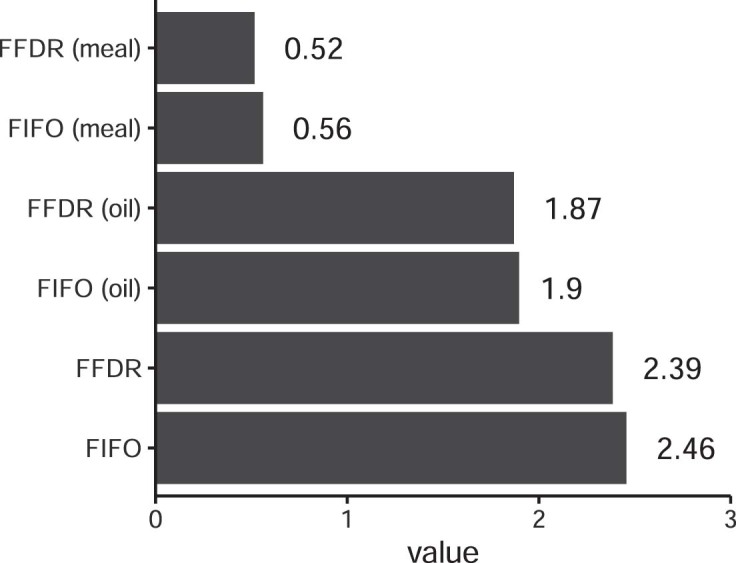
Historical FIFO metrics for assessing wild fish use in salmon production. Bars show values for FIFO (fish in: fish out) and forage fish dependency ratio (FFDR), for fish meal and fish oil separately and combined. FIFO is the amount of wild fish used in feed for production of one kg of farmed salmon. FFDR is equivalent to FIFO, but for fish meal and fish oil produced from whole forage fish only. These metrics involve ‘double counting’ of feed resources, assuming fishmeal and fish oil were generated separately.

For all ECM species, we extracted nutrient content data from Norwegian food composition tables^72^, focusing on nine nutrients that are essential in human diets and concentrated in seafood (calcium, iodine, iron, selenium, zinc, omega-3 fatty acids (EPA and DHA), vitamins A, D, B_12_) (Supplementary Table 3). Nutrient concentrations were multiplied by the total volume of edible whole fish required to produce fishmeal and fish oil (corrected by edible portion), giving the total volume of nutrients in ECM feed fish and in Atlantic salmon in 2020 (Supplementary Table 4). These values were used to estimate nutrient retention for each of the nine nutrients, at three Atlantic salmon edible portion sizes (58%, 73%, 88%) (Supplementary Table 5). We also assessed nutrient retention in the context of total FIFO and the FFDR using fishmeal and fish oil produced from whole fish only^73^. These metrics were estimated for fishmeal and fish oil combined, and separately for fish oil and fishmeal. Values were within the range of previously published values for Norway salmon (Extended Data Fig. 4)^73^, but do not account for additional fish production from unused fishmeal (for example, eFIFO^23^).

We next visualized the potential nutritional value of ECM feed fish and farmed Atlantic salmon by estimating the contribution of an edible portion to nutrient reference values (NRVs), extracted from UK dietary reference values^62,74^. For each nutrient estimated above (Supplementary Table 3), we extracted the recommended daily nutrient intake for adult women (females aged 19–64 years old). We used WHO and FAO guidelines^75^ for omega-3 (EPA and DHA) intake of 0.25 g d^−1^, as this nutrient is not currently included in UK guidelines. We used these reference limits to identify potential contributions of feed fish and farmed salmon to nutrient intakes of UK consumers. First, for each feed species and for Atlantic salmon, we estimated the portion size required to reach the recommended nutrient intake. Second, for the average feed fish and for Atlantic salmon, we estimated the contribution of 140 g of raw tissue (recommended seafood portion in the United Kingdom^76^) to recommended nutrient intakes. These two metrics were used to contrast the nutrient content of ECM feed fish and that of farmed salmon in the context of dietary requirements of UK consumers.

We then examined changes in nutrient retention, seafood production, fish oil requirements and by-product production associated with allocating different amounts of edible whole fish for direct human consumption. We developed simulations that removed edible whole fish from fish oil only (based on Norwegian volumes in 2020) and allocated these for direct consumption. In each simulation, we also estimated the total seafood produced, accounting for edible portions of salmon and forage fish, increasing incrementally from 0% of whole fish consumed directly (that is, business as usual) to 100% of whole fish consumed directly. Simulations were run using values for all edible whole fish used in fish oil, and separately for each edible whole-fish species (blue whiting, herring, mackerel, Pacific anchoveta, Peruvian anchoveta, sprat). We used these simulations to recalculate nutrient retention across all edible species and to estimate additional seafood production, additional fish oil (or novel alternatives) required from new sources to replace consumed whole fish (maintaining salmon production at 2020 levels) and the volume of new by-products created from consumed whole fish.

We assessed current intake of edible feed fish species and farmed salmon, using National Diet and Nutrition Survey data. Annually, a representative sample of around 500 adults (aged 19+ years) and 500 children (aged 1.5–18 years) complete a 4 day food diary, which includes information on seafood species consumed. We extracted all participants that reported consumption of food products containing salmon, mackerel, herring, anchovy or whiting in 2019. Average portion sizes (g) and the proportion of respondents consuming each species were combined with UK population census data for 2019^77^ to estimate the annual consumption (g per capita per year) of each species by adults and children.

### Statistics and reproducibility

No statistical method was used to predetermine sample size. No data were excluded from the analyses. The experiments were not randomized. The investigators were not blinded to allocation during experiments and outcome assessment. R version 4.3 was used for analysis, and the script is available as indicated in Code Availability.

### Reporting summary

Further information on research design is available in the Nature Portfolio Reporting Summary linked to this article.

### Supplementary information

Reporting Summary

Supplementary Data 1 Supplementary Tables 1–5.

### Source data

Source Data Fig. 1 Feed fish species quantity and nutrient data.

Source Data Fig. 2 NRV data for different nutrients across fish species.

Source Data Fig. 4 Edible nutrient retention data and scenario data.

## Data Availability

All data are available at https://github.com/jpwrobinson/SalmonFeedNutrients. Source data are provided with this paper.

## Extended data

>

## Code availability

All code is available at https://github.com/jpwrobinson/SalmonFeedNutrients.
